# Supplementary material for: Balance dysfunction the most significant cause of in-hospital falls in patients taking hypnotic drugs: A retrospective study
Source: PLoS One. 2022 Sep 1;17(9):e0272832. doi: 10.1371/journal.pone.0272832 (PMC9436085; doi:10.1371/journal.pone.0272832)
Supplement: S1 Table — (DOCX) [file pone.0272832.s001.docx]

Table● . Cox regression analysis for fall

| Factors | Univariate analysis for fall OR  (95% Confidence interval, P-value) | Multivariate analysis for fall OR  (95% Confidence interval, P-value) |
| --- | --- | --- |
| Benzodiazepines drugs | 1.21  (0.76-1.89, 0.4024) | 1.50  (0.94-2.39, 0.089) |
| Z-drugs | 0.57  (0.42-0.75, <.0001) | 0.69  (0.52-0.93, 0.0135) |
| Melatonin receptor agonists | 1.01  (0.68–1.50, 0.9541) | 0.78  (0.52-1.18, 0.24) |
| Orexin receptor antagonists | 1.76  (1.31–2.37, 0.0002) | 1.28  (0.94-1.74, 0.1128) |

Multiple logistic regression adjusted age, sex, dosage, standing test for Imbalance and disequilibrium levels, the use of multiple drugs, angiotensin converting enzyme inhibitors, alpha blockers, beta blockers, alpha beta blockers, loop diuretics, and selective serotonin reuptake inhibitors.

Abbreviations: OR; odds ratio.

Supplementally table1: Cox regression analysis for falls in patients who took orexin receptor antagonists

| Factors | OR  (95% Confidence interval, P-value) |
| --- | --- |
| age | 1.01  (0.10–1.03, 0.1198) |
| Balance disorder | 5.41  (2.87–10.19, <.0001) |

Multiple logistic regression adjusted age, standing test for Imbalance and disequilibrium levels
