# Supplementary material for: Balance dysfunction the most significant cause of in-hospital falls in patients taking hypnotic drugs: A retrospective study
Source: PLoS One. 2022 Sep 1;17(9):e0272832. doi: 10.1371/journal.pone.0272832 (PMC9436085; doi:10.1371/journal.pone.0272832)
Supplement: Attachment — Submitted filename: PLOS one SIDE Responses to REVIEWER 2 final.docx [file pone.0272832.s002.docx]

Responses to REVIEWER 2:

Thank you for your comments regarding our manuscript (PONE-D-22-02308). We appreciate your comments, which have helped us to improve our manuscript. In line with your comments, please find below our point-by-point responses.

Specific comments

Introduction

**Comment 1:**

Please describe that why patients with a hospital-acquired hip fracture have higher complication rate than those who fall outside of the hospital.

**Answer:** We fully agree with your comment. We added the reason why the patients with a hospital-acquired hip fracture have a poor prognosis. The patients with a hospital-acquired hip fracture are already in poor condition physically and mentally and have an especially poor outcome [1]. We added this sentence in the introduction sections. Thank you for your advice.

(Page4, line83-85)

**Comment 2:** The study gap is not clearly stated in the introduction section. The authors can highlight the components of the standing balance postural controls in elderly subjects.

**Answer:** We apologize for our unclear description. We should have clarified the study gap in the introduction section. Hypnotic drugs, including z-drug and orexin receptor antagonists, affect balance ability [2]. Therefore, we need to pay attention to the balance function of patients taking hypnotic drugs. We revised the introduction section to clarify this study’s aim in the introduction sections.

(Page5, line111-114)

**Comment 3:** o There is a discrepancy in the duration of data gathering. In line 122, the authors stated that data gathering was conducted from July to December 2021. However, in line 128 (for figure legend), it was reported as July to December 2020. Please check that which one is correct.

**Answer:** We apologize for the typo. I wrote the following sentence. Thank you for your careful reading. We did a retrospective study and enrolled all hospitalized patients who were taking hypnotic drugs from July to December 2020 at Kurume University Hospital.

(Page5, line126-128)

**Comment 4:** In introduction section, the authors stated that in their hospital benzodiazepine in not prescribed for patients. However, in method section the benzodiazepine is one of the hypnotic drugs that prescribed to the patients.

**Answer:** Thank you for pointing out the unclear points in our sentences. Our hospital recommends that physicians not prescribe benzodiazepines in case of insomnia. However, there were patients who had been taking benzodiazepines before they were admitted to our hospital in this study. As you point out, the introduction section seems misleading. We removed this sentence and revised the introduction sentences.

**Comme**nt 5: Line 204: please report the p-value for fall ratio between z-drugs and melatonin receptor agonists.

**Answer:** Thank you for your careful reading. As you suggested, we wrote the p-value for fall ratio between z-drugs and melatonin receptor agonists. We added the following sentence in the results sections.

However, there was no significant difference in the fall ratio between z-drugs and melatonin receptor agonists (p=0.1352).

(Page9, line213-214)

**Comment 6:** Please compare the results of this study with related articles that published previously.

**Answer:** Thank you for your advice. We compared the results of each drug fall rate in this study with the previous study in the discussion section. I appreciate your valuable comments.

(Page12, line300- Page13, line315)

**Comment 7:** In limitation section, please describe that why retrospective nature of the study design is one of limitation of the study.

**Answer:** Thank you for pointing this out. Our study evaluated balance function in patients who took the hypnotic drug. We assessed patients' balance ability at admission in this study. On the other hand, the timing of hypnotic prescriptions varies. Some patients had begun taking hypnotics before being admitted, while others started them during hospitalization. This is a limitation of this retrospective study. A prospective randomized controlled trial is needed to resolve this issue. We have added this point to the limitations section.

(Page14, line356-364)

**Comment 8:** **- Conclusion: Relevant to the main findings.**

**Answer:** We appreciate your comments. As you pointed out, the conclusion was inadequate. We modified the conclusion according to the results as follows.

This study investigated the ratio of patients who took the hypnotic drug. The falls ratio for z-drugs was significantly lower than for benzodiazepine drugs, and orexin receptor antagonists. Moreover, we also investigated the factors associated with falls. Balance disorder was an independent factor for falls using decision-tree analysis. Therefore, a physician should consider not only the choice of drugs but also balancing functions in inpatients.

(Page14, line368-373)

**Comment 9: General point: There is some grammatical error in the text that should be revised by a native fluent reviewer.**

**Answer:** Thank you for your advice. A native speaker had checked this manuscript before submission. We asked her to recheck this manuscript after revision. We consider that this manuscript's grammar has improved.

1.	Foss NB, Palm H, Kehlet H. In-hospital hip fractures: prevalence, risk factors and outcome. Age Ageing. 2005;34(6):642-5. Epub 2005/11/04. doi: 10.1093/ageing/afi198. PubMed PMID: 16267194.

2.	Bland H, Li X, Mangin E, Yee KL, Lines C, Herring WJ, et al. Effects of Bedtime Dosing With Suvorexant and Zolpidem on Balance and Psychomotor Performance in Healthy Elderly Participants During the Night and in the Morning. J Clin Psychopharmacol. 2021;41(4):414-20. Epub 2021/06/29. doi: 10.1097/JCP.0000000000001439. PubMed PMID: 34181362.
