# Supplementary material for: Balance dysfunction the most significant cause of in-hospital falls in patients taking hypnotic drugs: A retrospective study
Source: PLoS One. 2022 Sep 1;17(9):e0272832. doi: 10.1371/journal.pone.0272832 (PMC9436085; doi:10.1371/journal.pone.0272832)
Supplement: Attachment — Submitted filename: PLOS one SIDE Responses to REVIEWER 3.docx [file pone.0272832.s003.docx]

Responses to REVIEWER 3:

Thank you for your comments regarding our manuscript (PONE-D-22-02308R1). We appreciate your comments, which have helped us to improve our manuscript. In line with your comments, please find below our point-by-point responses.

Specific comments

**Comment 1:**

All the reviewers’ comments are addressed. However, I would like to ask about figure 6 is balance disorder " no" when the level of SIDE is:2a,1,0?

**Answer:** I apologize for my miswriting in figure 6. We defined patients who were unable to do tandem standing as having balance disorder (SIDE levels 0, 1, and 2a) (Page6, line153-155). We revised figure 6, thank you for your careful reading.

**Comment 2:** Also, the aim of work in abstract: The aim of this study was to investigate the cause of falls in hospitalized patients taking hypnotic drugs, while, in the introduction section, The purpose of this study was to investigate the fall rate of each patient who took the hypnotic drug and the factor associated with falls. In the conclusion section, "This study investigated the ratio of patients who took the hypnotic drug" is this accurate as regards the results?.

**Answer**: As you pointed out, the aim in the abstract and introduction was not appropriate. I revised the aim in the abstract section as follows. “The purpose of this study was to investigate the fall rate of each patient who took the hypnotic drug and the factor associated with falls.” (Page3, line51-53) Moreover, I miswrote the conclusion in the main text. I revised the conclusion as follows.” This study investigated the fall ratio of patients who took the hypnotic drug. The fall ratio for z-drugs was significantly lower than for benzodiazepine drugs, and orexin receptor antagonists. Moreover, we also investigated the factors associated with falls. Balance disorder was an independent factor for falls using decision-tree analysis. Therefore, a physician should consider not only the choice of drugs but also balance functions in inpatients.” (Page14, line369-374) I appreciate your valuable comments.
